# Supplementary figures and images for: Identification by shape-based virtual screening and evaluation of new tyrosinase inhibitors
Source: PeerJ. 2018 Jan 26;6:e4206. doi: 10.7717/peerj.4206 (PMC5788061; doi:10.7717/peerj.4206)

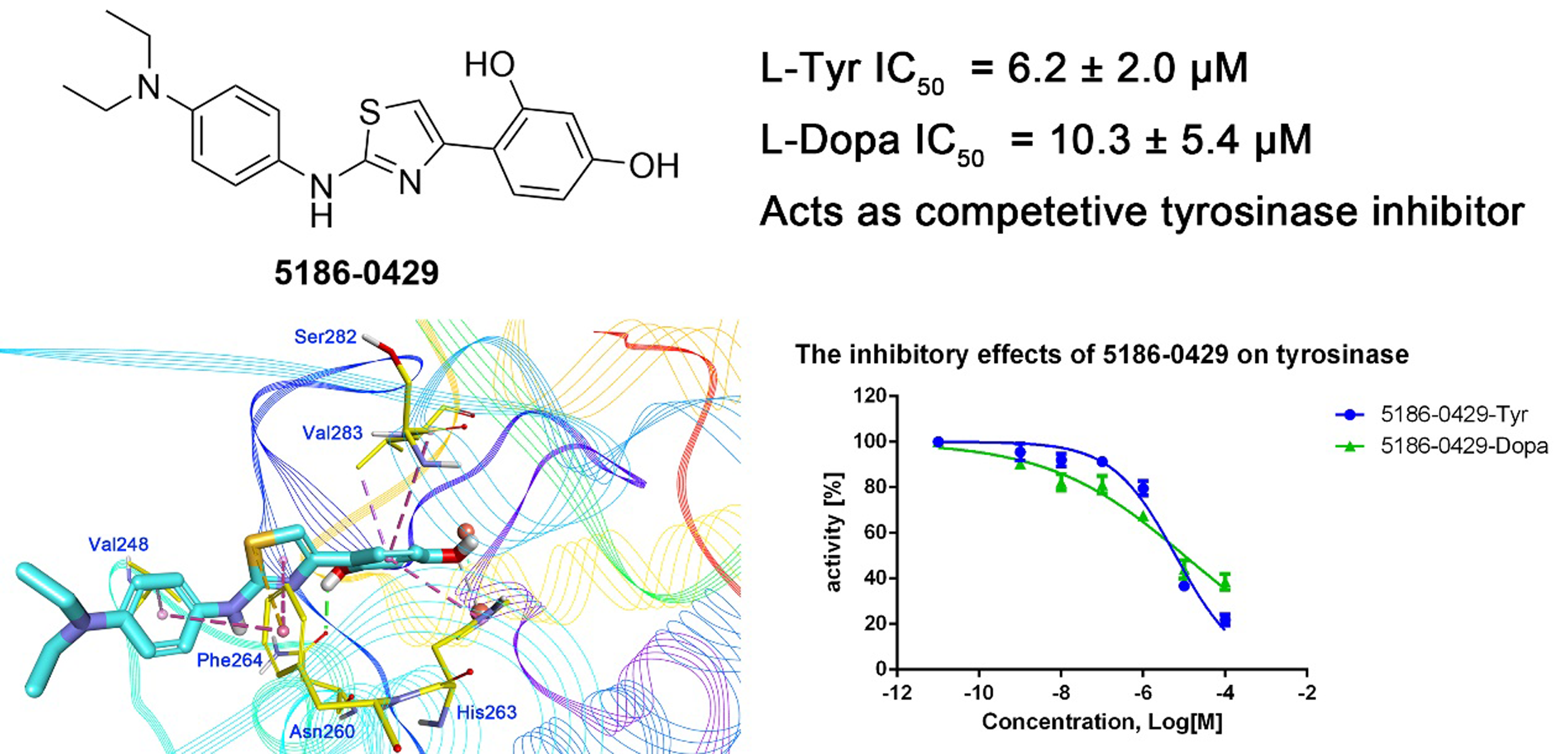

Supplement: Supplemental Information 6 [file peerj-06-4206-s006.png]
